# Endoclip combined with colonic transendoscopic enteral tubing: a new approach for managing iatrogenic colonoscopy perforation

**DOI:** 10.1007/s00464-024-10671-8

**Published:** 2024-01-29

**Authors:** Xiaomeng Jiang, Chunhua Ni, Fatema Tabak, Yi Li, Faming Zhang

**Affiliations:** 1 Department of Gastroenterology, Sir Run Run Hospital, Nanjing Medical University, 109 Longmian Avenue, Nanjing, 211166 China; 2 Department of General Surgery, Jiangning District Hospital of Traditional Chinese Medicine, 657 Tianyin Avenue, Nanjing, 211199 China; 3 Department of Microbiota Medicine & Medical Center for Digestive Diseases, The Second Affiliated Hospital of Nanjing Medical University, 121 Jiang Jia Yuan, Nanjing, 210011 China; 4 Department of Microbiota Medicine, Sir Run Run Hospital, Nanjing Medical University, 109 Longmian Avenue, Nanjing, 211166 China

**Keywords:** Iatrogenic colonoscopy perforation, Transendoscopic enteral tube, Endoclip, Complication

## Abstract

**Background:**

Iatrogenic colonoscopy perforation (ICP) is a rare but most serious complication during colonoscopy investigation. However, endoscopic closure plays an important role in the dealing with ICP with the development of endoscopic techniques presently, there are still some portion of patients transferred to surgery.

**Methods:**

Once a perforation was detected, endoclips were used to closed the defect of the colon. Then a colonic TET was planted inside the colon. The terminal end of the TET was put proximally to or near the location of the perforation. Then gas and fluid was sucked out through the TET with a syringe every 4 h.

**Results:**

Three cases were treated with endoclip closure and colonic TET drainage. Case 1 was caused by urgent immediate perforation during routine colonoscopy, case 2 was delayed perforation after snare resection, and case 3 was ESD-related perforation. All patients got healed, no one transferred to surgery.

**Conclusions:**

A combination of endoclip closure and colonic TET drainage might be an easy and potential method in the dealing with different types of ICP. This study may offer a novel paradigm for addressing endoscopy-related intestinal perforations.

Iatrogenic colonoscopy perforation (ICP) is the most serious complication that could develop during colonoscopy, despite its rarity. Peritonitis and even sepsis may occur if it goes undetected and untreated in time, which would imply a higher mortality rate [1]. The incidence of ICP ranges from 0.03 to 0.8% in diagnostic colonoscopy procedures and from 0.3 to 3% in therapeutic colonoscopy procedures, respectively [2]. Once perforation occurs, the surgical intervention is usually recommended. However, the therapeutic outcomes of endoscopic closure have been improved with the development of endoscopic technology, there are still some risk in transferring to surgery in patients treated with endoscopic closure. Endoscopic closure techniques mainly include endoclips, over-the-scope clip (OTSC) system, band ligation and endoloop. Each has its advantages and disadvantages and should be selected according to the location, type, and size of the iatrogenic perforation as well as the endoscopist’s experience [3, 4]. In this report, we describe a new endoscopic approach for managing ICP, which involved a combination of endoclip closure and colonic transendoscopic enteral tube (TET) drainage. Our results indicate successful outcomes with the use of this new method for managing three reported cases of ICP.

## Methods

**Fig. 1:**
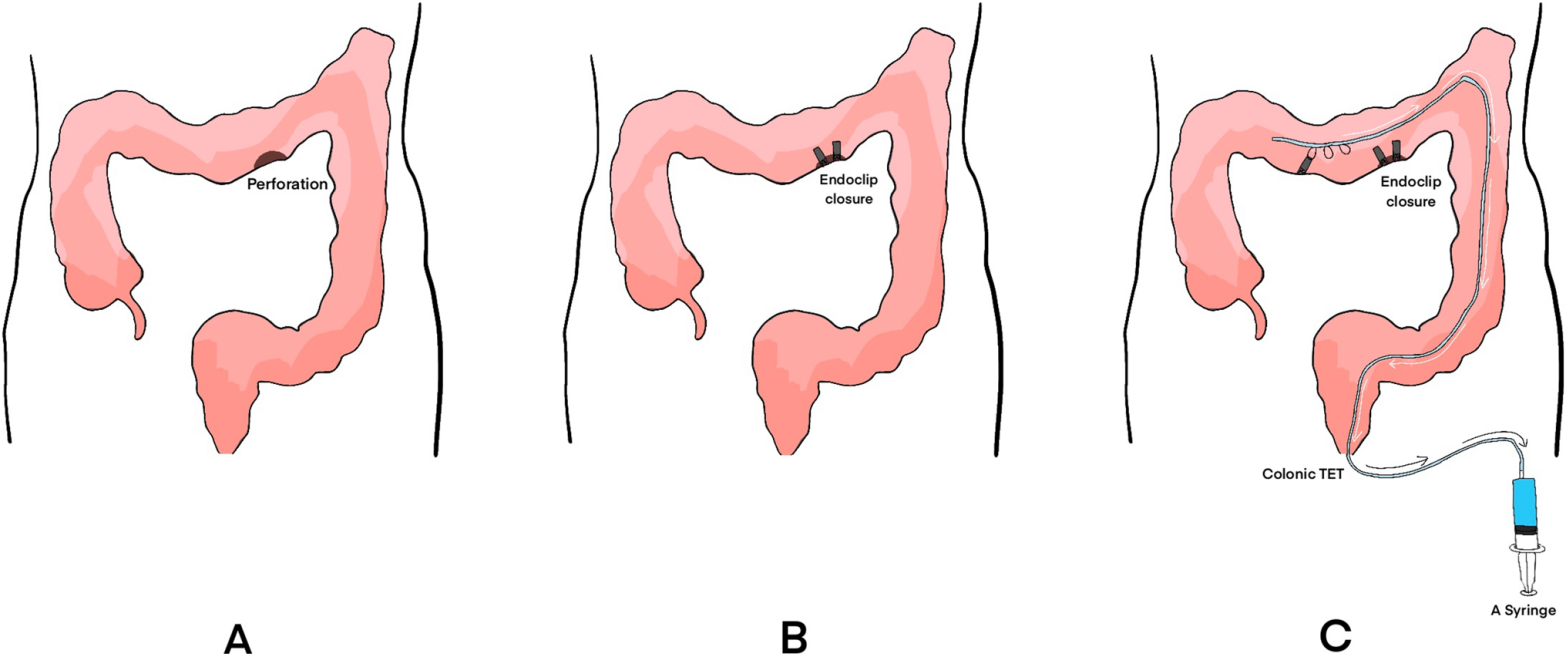
**A** Perforation detected. **B** Endoclips applied to close the perforation. **C** Colonic TET was inserted into the colon after perforation closed with endoclips. Gas and fluid was sucked with a syringe though the TET

**Fig. 2:**
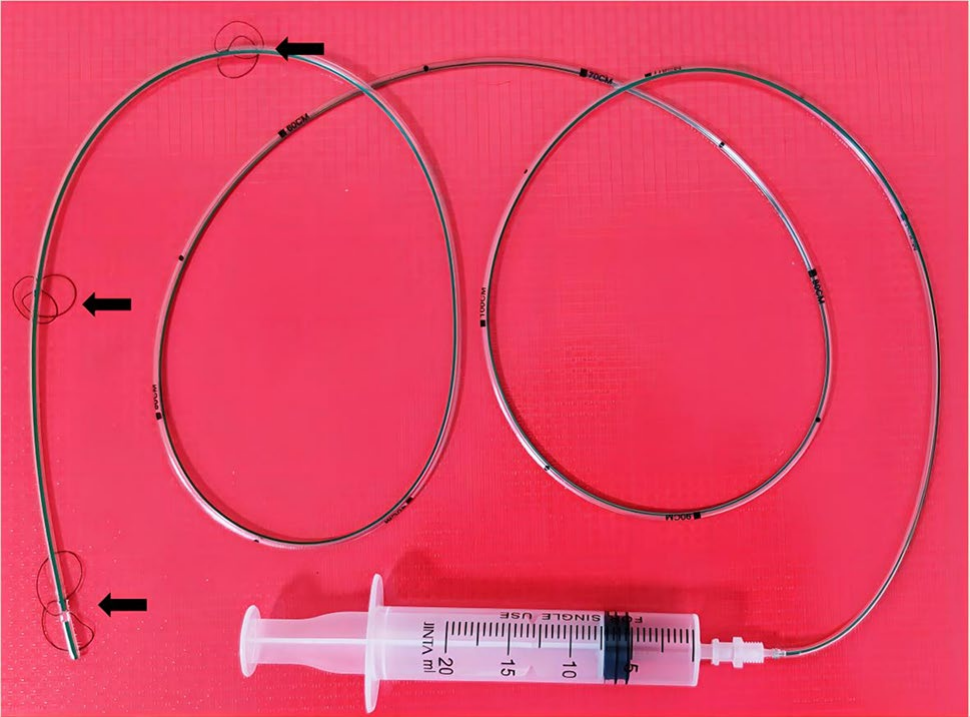
The colonic TET is showed in the figure, with an outer diameter of 2.7mm. The type is FMT-DT-F-27/1350. Three separate loops are designed on the terminal end of the tube, which contribute to the tube fixed to the colonic mucosa with endoclips (black arrow). Side-holes are also designed on the terminal end of the tube which help to drainage out gas and fluid

Three cases with ICP were collected retrospectively. Once a perforation was detected, endoclips (ROCC-D-26-230-C, Micro-Tech, Nanjing, China) were used to closed the defect of the colon. Then a colonic TET (Diameter 2.7 mm, FMT-DT-F-27/1350, FMT medical, Nanjing, China) was planted inside the colon. The terminal end of the TET was put proximal to or near the location of the perforation. Then gas and fluid was sucked out through the TET with a syringe every 4 h (see Fig. 1 A–C). More details about the colonic TET were described in Fig. 2. How to implant the colonic TET was shown in the video published by our team previously [5]. Given the retrospective study design, this study was not registered in a public trial registry, and IRB approval and written consent was not applied for. The signed consent of implantation of the colonic TET as a regular procedure in China was obtained from the patients.

## Case presentation

### Case 1

**Fig. 3:**
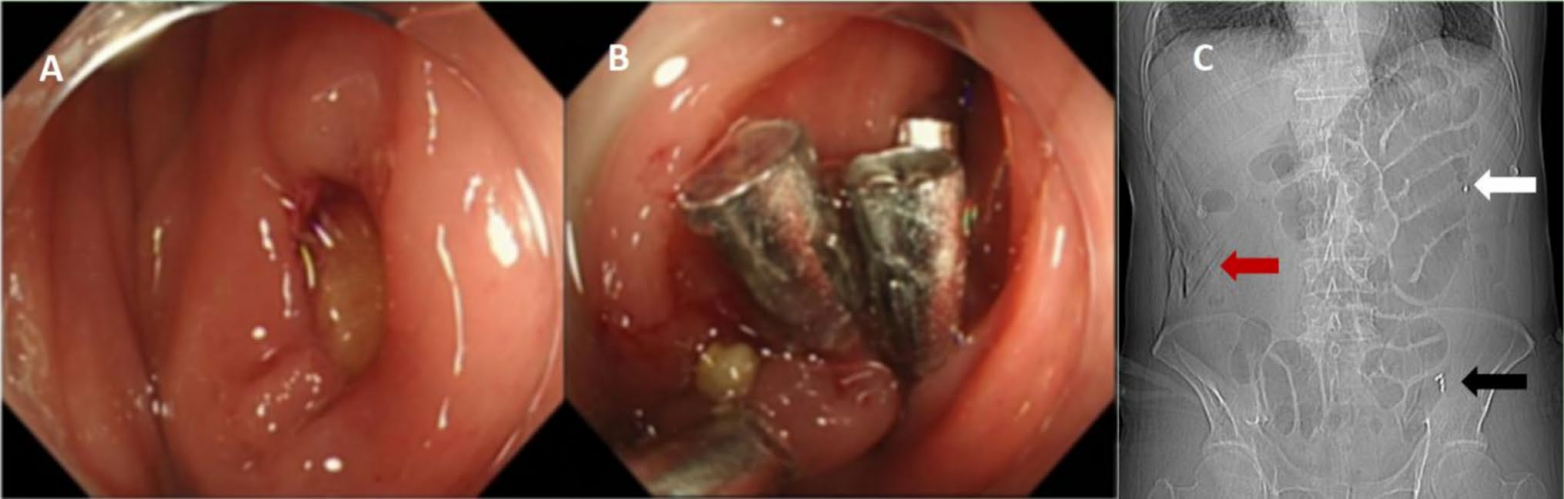
**A** Perforation in sigmoid colon. **B** Endoclips were applied to close the perforation. **C** Colonic transendoscopic enteral tube was inserted into the colon after perforation closed with endoclips. (Red arrow: Free gas was visible around the liver. White arrow: Endoclip was used to fix the colonic TET to the mucosa of the splenic flexure of the colon. Black arrow: Endoclips used to close the perforation)

A 59-year-old man underwent colonoscopy due to diarrhea. The patient experienced worsening abdominal distention and pain during the procedure, and a subsequent 15 mm diameter perforation was identified in the sigmoid colon. Four endoclips were used to close the perforation successfully. And then, a colonic TET was inserted into the colon, proximal to the location of the closed perforation and fixed to the mucosa of the colonic splenic curvature with one endoclip (see Fig. 3 A–C). The patient received supportive care, including fasting, intravenous fluids, and antibiotics after the procedure. The colonic TET was used to suction gas and fluid by a syringe from the site of the perforation as much as possible every 4 h. The patient's abdominal pain and body temperature gradually improved. Within 72 h of the surgery, the patient's temperature (up to 38.4 °C) returned to normal. Serum procalcitonin levels (up to 10.94 ng/ml, normal 0.05 ng/ml) declined within several days after the procedure. The patient had the colonic TET removed and was able to resume oral feeding on the fifth day and was discharged on the ninth day after the procedure.

### Case 2

**Fig. 4:**
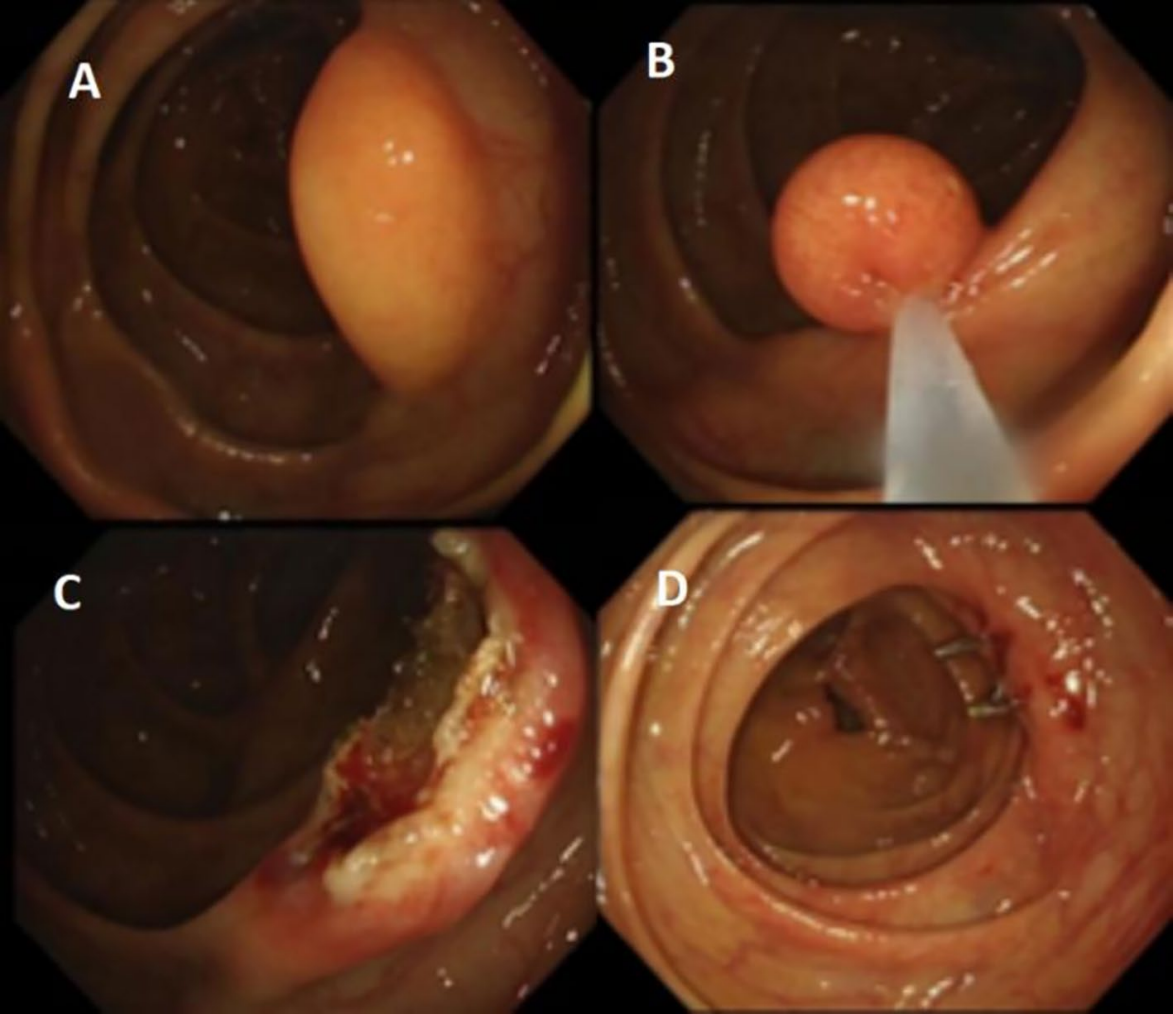
**A**–**D** Snare resection of submucosal lipoma in the ascending colon

**Fig. 5:**
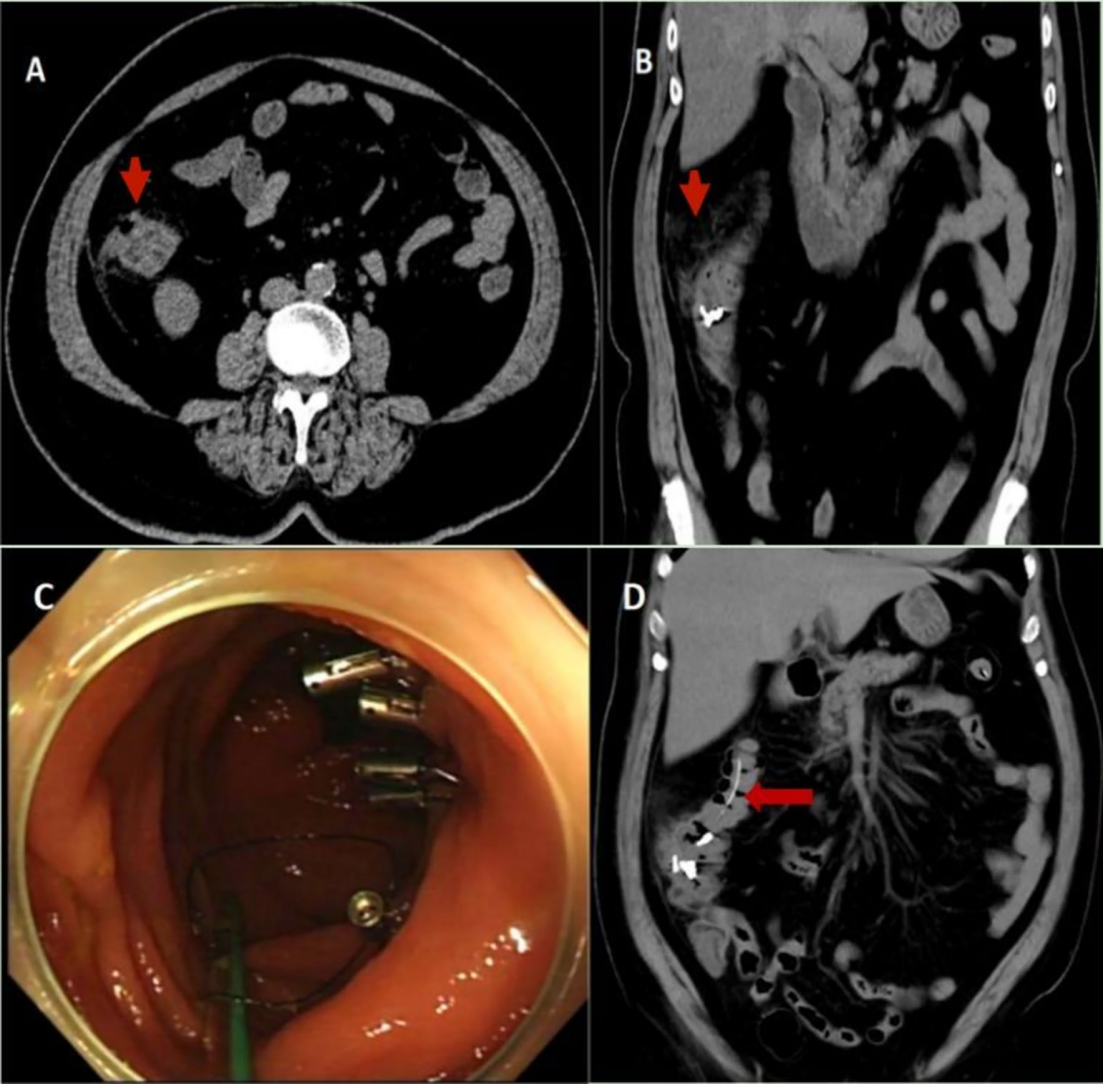
**A**–**B** Abdominal CT shows gas and inflammatory exudation around the ascending colon (red arrow). **C** Two additional endoclips applied to secure the wound, the colonic TET placed and fixed near the wound. **D** Follow-up CT showed exudation getting better after TET drainage (red arrow: colonic TET)

A 58-year-old woman was diagnosed with a submucosal lipoma with a diameter of about 20 mm in the ascending colon during a routine colonoscopy. Snare resection was undertaken to excise the lesion and three endoclips were used to seal the surgical wound. There was no perforation during the procedure, and the surgical site remained intact. (Fig. 4 A−D). About 8 h later, the patient complained of abdominal pain, initially in the right lower abdomen and gradually spreading throughout the whole abdomen. The pain severity worsened and was accompanied by chills and fever, with the peak body temperature reaching 39.2 °C. Physical examination revealed positive peritoneal irritation. An abdominal CT scan revealed gas in the colonic wall and fluid exudation outside the colon, indicating a delayed colonic perforation (see Fig. 5 A–B). A follow-up colonoscopy was performed about 24 h later, and two additional endoclips were used to further secure the surgical wound. After that, a colonic TET was inserted and fixed to the colonic mucosa around the wound with one endoclip (see Fig. 5 C−D). Fasting, intravenous fluids and antibiotics were administered as in the case 1. Suction of air and fluid via the colonic TET was also undertaken every 4 h. Abdominal pain and body temperature returned to normal 48h after the second colonoscopy. The elevated white blood cell count (up to 32.45 × 10^9^/l, normal 3.5−9.5 × 10^9^/l) and C-reaction protein level ( CRP, up to 165.61 mg/l, normal 0−10 mg/l) both gradually declined to the normal levels. Oral feeding was resumed and the colonic TET was removed on the fifth day. The patient was discharged on the seventh day after the second procedure.

### Case 3

**Fig. 6:**
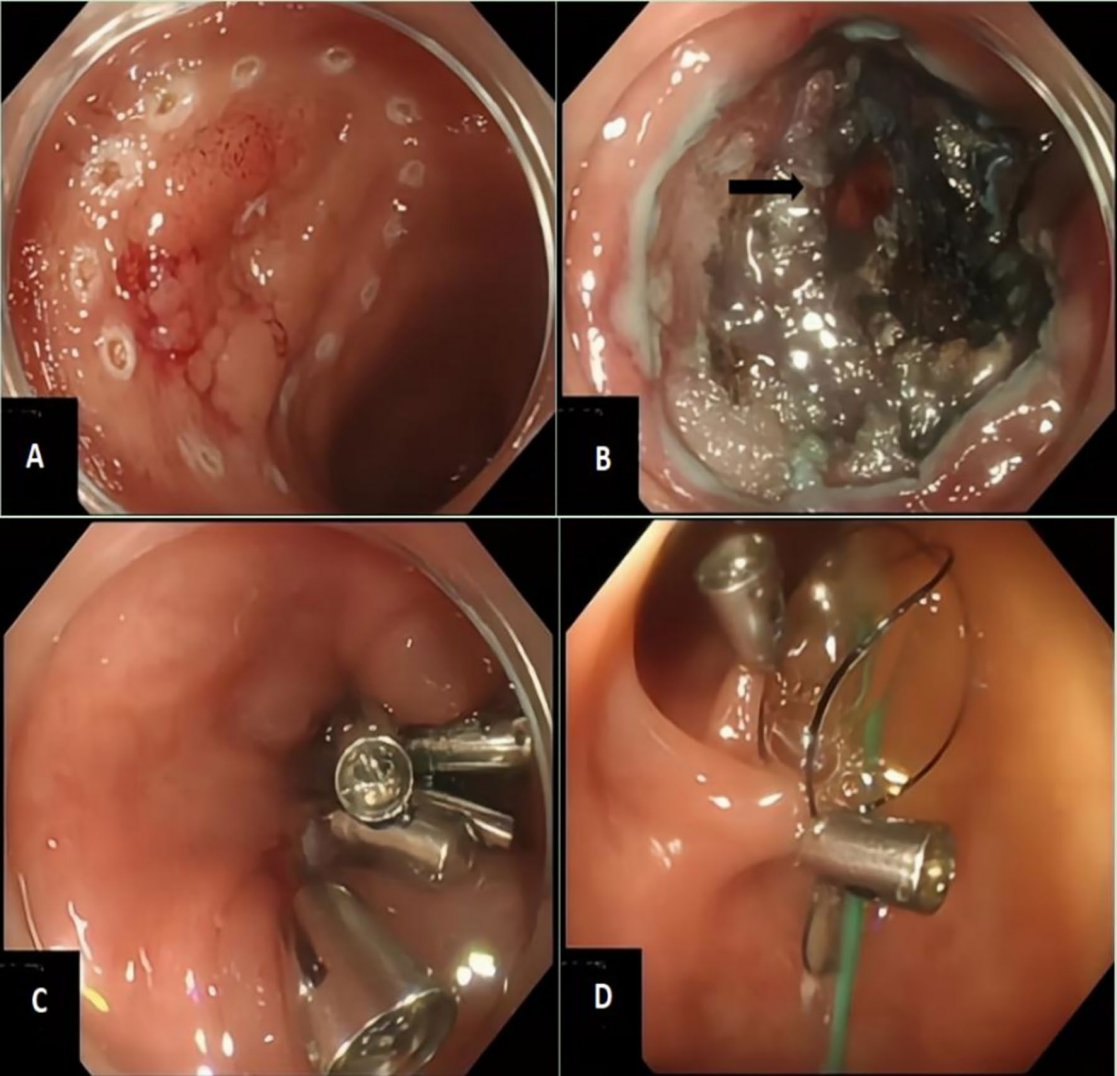
**A-C** Laterally spreading tumor excised by ESD. Black arrow: perforation in the ESD wound. **D** Colonic TET inserted and fixed near the hepatic curvature

A 58-year-old woman underwent an endoscopic submucosal dissection (ESD) for a 15 × 20 mm laterally spreading tumor (LST) in the sigmoid colon. An 10mm perforation in the ESD site was detected following the en bloc tumor resection. Five endoclips were used to close the wound; then, a TET tube was inserted and fixed to the colonic mucosa near the hepatic flexure with two endoclips (see Fig. 6). Post-operation management was similar to case 1 and 2. The patient’s symptoms and WBC were normal, so the oral feeding was resumed and the colonic TET was withdrawn on the third day. The patient was discharged on the fifth day after the procedure.

## Literature review and discussion

Colon perforation is defined by a complete tear in all four layers of the colon (mucosa, submucosa, muscularis propria, and serosa), resulting in pneumoperitoneum, fecal peritonitis, and sepsis [3]. Although it is rare, perforation can be one of the most serious complications related to the colonoscopy [6]. Recently, with the number of screening, diagnostic, and therapeutic colonoscopies performed annually increasing worldwide, ICP is becoming more frequent, which physicians should be paying more attention to [7, 8]. A recent retrospective study in 2022 showed that the incidence of ICP was 0.09%, with 0.06% occurring during diagnostic colonoscopies, and 0.25% during therapeutic colonoscopies [9].

Many risk factors and different mechanisms have been reported in research related to ICP. These risk factors include elder age, female gender, low body mass index, low albumin level, comorbidities, diverticulosis, Crohn's disease, undergoing therapeutic technique, and endoscopist experience [10]. Other risk factors include aggressive advancement or angulation of the colonoscope, improper pressure on the colonic wall, excessive air insufflation, and improper application of electrocautery during therapeutic procedures (polypectomy, EMR, and ESD) [3, 6, 9]. In this report, the specific cause of the colon perforations was identified for each of the three cases. The perforation in case 1 was due to direct trauma from aggressive advancement in a difficult procedure maneuvered by an inexperienced endoscopist. Case 2 resulted from electro-coagulative damage to the muscularis propria and lake of submucosal injection before snare resection. While case 3 was due to the insufficient submucosal injection, the large size of the LST and cutting too deep during the ESD procedure. Early identifying of the risk factors and taking corresponding measures can potentially reduce the incidence of colon perforation during colonoscopy.

The endoclip was initially used to close the ICP by Yoshikane in 1997 and has recently been considered beneficial and effective in sealing the perforation and avoiding surgery in most cases [10]. For minor perforations (less than 10 mm), endoclip closure of ICP is usually recommended, with a success rate of 59–100% [11]. Nevertheless, certain cases need to be converted to surgery (4.5–25.8%), although the clipping closure was theoretically successful during the procedure [6, 12]. After endoclips replacement, it is difficult to determine whether the closure is complete. Animal models showed that endoclips might create satisfied apposition of the mucosa and submucosa, whereas that apposition of the muscularis propria and serosa was not possible due to the superficial bite of the clips [13].This kind of superficial closure may increase the risk of delayed small leakage of fecal fluid, leading to delayed peritonitis, as mentioned in case 2. Further measures should be taken to improve the technical success rate of endoclip closure and reduce the possibility of conversion to surgery.

Long-term nasogastric tube (NGT) placement has been suggested and practiced in managing of upper gastrointestinal perforations, with the function of continuously absorbing upper gastrointestinal fluid [14]. However, with colonic perforations, no comparable tube could be placed within the colon, maintaining long-term aspiration of colonic contents until the innovation of a colonic TET by Faming Zhang in 2014 [15]. With three separate loops on the terminal segment of the colonic TET, it could be fixed to the colonic wall with one to four clips and maintained within the colon for one to two weeks for most patients (few could be maintained four weeks) [5]. The initial function of this device is to meet the needs of patients requiring multiple washed microbiota transplantation (WMT) or the whole-colon administration of medications within one to two weeks [16]. Furthermore, colonic gas or fluid can be aspirated through the colonic TET with a syringe or a negative pressure bag, which may help decrease the pressure inside the colon lumen and the volume of fluid leaking out of the colon in the case of perforation.

Two delayed perforation cases, one with Crohn's disease perforated after endoscopic balloon dilation and another with ulcerative colitis and laterally spreading mild dysplasia after ESD, were both successfully treated with colonic TET draining alone, as reported by Zhang et al. [17]. Based on our previous experience, we combined using endoclips and colonic TET to manage different types of colonic perforation, including urgent immediate perforation in case 1, delayed perforation in case 2, and ESD-related perforation prevented from getting worsen in case 3. All of them got satisfied outcomes within short time.

The over-the-scope clip closure and the endoloop-assisted clip closure was reported to successfully treat the colonic perforation previously [18, 19]. Though effective, these devices were not equipped in every hospital, and the operation procedure was very complicated, needing experienced endoscopists. However, endoclip combined with colonic TET seems to be easier to be obtained and applied.

Endoscopic treatment is usually recommended when the perforation site is identified within 4 h following the procedure [10]. However, in case 2, the second endoscopy procedure was performed 24 h following the initial procedure, which did not worsen the patient's condition. Besides the fact that this is the only case report implying this endoclips and TET combination, it also suggests the possibility of extending the time window of endoscopic treatment. In order to achieve a sufficient drainage effect, we propose that the terminal end of the colonic TET passes across the perforation site to facilitate draining as much gas and fluid from the proximal colon. If the tube cannot be indwelt across through the perforation site to the proximal colon, placing the end of the tube close to the perforation would also be effective.

## Conclusion

Endoscopic treatments have played a vital role in managing ICP, which is still challenging for many endoscopists. In this report, we present a new and easy method, the combination of endoclips closure and colonic TET drainage, for the treatment of different types of ICP, achieving satisfying clinical effects and avoiding the conversion to surgery. Our report may provide endoscopists with more confidence in treating perforations, reliable solutions, and improve doctor-patient satisfaction.

## References

[1] Cai SL, Chen T, Yao LQ, Zhong YS (2015). Management of iatrogenic colorectal perforation: from surgery to endoscopy. World J Gastrointest Endosc.

[2] Gündeş E, Çiyiltepe H, Aday U, Çetin DA, Senger AS, Uzun O (2017). Emergency cases following elective colonoscopy: Iatrogenic colonic perforation. Turk J Surg.

[3] Jung Y (2020). Endoscopic management of iatrogenic colon perforation. Clin Endosc.

[4] Paspatis GA, Dumonceau JM, Barthet M, Meisner S, Repici A, Saunders BP, Vezakis A, Gonzalez JM, Turino SY, Tsiamoulos ZP (2014). Diagnosis and management of iatrogenic endoscopic perforations: European Society of Gastrointestinal Endoscopy (ESGE) position statement. Endoscopy.

[5] Zhang T, Long C, Cui B, Buch H, Wen Q, Li Q, Ding X, Ji G, Zhang F (2020). Colonic transendoscopic tube-delivered enteral therapy (with video): a prospective study. BMC Gastroenterol.

[6] Thompson EV, Snyder JR (2019). Recognition and management of colonic perforation following endoscopy. Clin Colon Rectal Surg.

[7] von Karsa L, Patnick J, Segnan N, Atkin W, Halloran S, Lansdorp-Vogelaar I, Malila N, Minozzi S, Moss S, European Colorectal Cancer Screening Guidelines Working G (2013). European guidelines for quality assurance in colorectal cancer screening and diagnosis: overview and introduction to the full supplement publication. Endoscopy.

[8] Bibbins-Domingo K, Grossman DC, Curry SJ, Davidson KW, Epling JW, Garcia FA, Gillman MW, Harper DM, Kemper AR, Force USPST (2016). Screening for colorectal cancer: US preventive services task force recommendation statement. JAMA.

[9] Cha RR, Kim HJ, Lee CM (2022). Clinical characteristics and outcome of iatrogenic colonic perforation related to diagnostic vs. therapeutic colonoscopy. Surg Endosc.

[10] Yoshikane H, Hidano H, Sakakibara A, Ayakawa T, Mori S, Kawashima H, Goto H, Niwa Y (1997). Endoscopic repair by clipping of iatrogenic colonic perforation. Gastrointest Endosc.

[11] De'Angelis N, Di Saverio S, Chiara O, Sartelli M, Martínez-Pérez A, Patrizi F (2018). 2017 WSES guidelines for the management of iatrogenic colonoscopy perforation. World J Emerg Surg.

[12] Yang DH, Byeon JS, Lee KH (2010). Is endoscopic closure with clips effective for both diagnostic and therapeutic colonoscopy-associated bowel perforation?. Surg Endosc.

[13] Raju GS, Ahmed I, Xiao SY (2006). Controlled trial of immediate endoluminal closure of colon perforations in a porcine model by use of a ovelclip device (with videos). Gastrointest Endosc.

[14] Lee JH, Kedia P, Stavropoulos SN, Carr-Locke D (2021). AGA clinical practice update on endoscopic management of perforations in gastrointestinal tract: expert review. Clin Gastroenterol Hepatol.

[15] Peng Z, Xiang J, He Z, Zhang T, Xu L, Cui B, Li P, Huang G, Ji G, Nie Y, Wu K, Fan D, Zhang F (2016). Colonic transendoscopic enteral tubing: a novel way of transplanting fecal microbiota. Endosc Int Open.

[16] Zhong M, Buch H, Wen Q, Long C, Cui B, Zhang F (2021). Colonic transendoscopic enteral tubing: route for a novel, safe, and convenient delivery of washed microbiota transplantation in children. Gastroenterol Res Pract.

[17] Zhang F, Wen Q, Cui B (2022). Drainage via colonic transendoscopic enteral tubing increases our confidence in rescuing endoscopy-associated perforation. Endoscopy.

[18] Russo S, Grande G, Manta R, Mangiafico S, Bertani H, Pigò F, Conigliaro R (2021). Large iatrogenic sigmoid colon perforation treated with endoloop-assisted clip closure and over-the-scope clip: a case report. Endoscopy.

[19] Nicolaou P, Velegraki M, Arna D, Psistakis A, Bachlitzanakis E, Flamourakis M, Paspatis GA (2022). Iatrogenic colonic perforation closure with an over-the-scope clip applied with a gastroscope 4 hours after index colonoscopy. Endoscopy.

